# Characteristics of cataracts in dry-hot valley-type high-altitude surgical cohort: a retrospective descriptive case series of 556 eyes from western China

**DOI:** 10.3389/fmed.2026.1921180

**Published:** 2026-09-23

**Authors:** Cheng Zuo, Zhaxi Ciren, Ya-Li Yang, Jun-Feng Yang

**Affiliations:** 1 Department of Ophthalmology, The Third People’s Hospital of Chengdu, Southwest Jiaotong University Affiliated Hospital, Chengdu, Sichuan, China; 2 Department of Ophthalmology, Derong County People’s Hospital, Derong, Sichuan, China

**Keywords:** cataract subtypes, dry–hot valley, high-altitude, surgical cohort, ultraviolet radiation

## Abstract

**Objective:**

To describe the morphological characteristics and severity distribution of age-related cataracts in a surgical cohort from a dry-hot valley-type high-altitude region, and to explore age- and gender-related patterns.

**Methods:**

This retrospective descriptive case series included 330 consecutive patients (556 eyes) who met cataract surgical criteria at the People's Hospital of Derong from October 1, 2023, to June 20, 2025. Cortical, nuclear, and posterior subcapsular opacities were graded using the Lens Opacities Classification System III (LOCS III). Univariable comparisons (chi-square, Mann–Whitney U) and multivariable logistic regression with cluster-robust standard errors were performed to identify factors associated with severe opacities. A sensitivity analysis using one randomly selected eye per patient was conducted. No individual UV exposure data were collected.

**Results:**

Cortical opacity had the highest mean grade (3.18), followed by posterior subcapsular opacity (2.64) and nuclear (2.59). Severe opacities (C4-5, N4-5, P3-4) accounted for 35.3%, 16.7%, and 60.4% of all examined eyes, respectively. Among eyes from patients aged ≤60 years, who made up 17.3% of the total sample, 63.5% had severe posterior subcapsular opacity, and 27.1% of this younger subgroup had mature cataracts (C5 or P4 with absent fundus red reflex). Age was significantly correlated with all three opacity types (Kendall's tau-b: cortical 0.336, *P* < 0.001; nuclear 0.432, *P* < 0.001; PSC 0.187, *P* = 0.004). Multivariable regression identified age (OR per year = 1.079, 95% CI: 1.051–1.107, *P* < 0.001) and diabetes (OR = 2.313, 95% CI: 1.149–4.656, *P* = 0.019) as independent risk factors for severe cortical opacity. Age was also independently associated with severe nuclear opacity (OR = 1.101, 95% CI: 1.067–1.136, *P* < 0.001) and severe posterior subcapsular opacity (OR = 1.035, 95% CI: 1.011–1.059, *P* = 0.004). No significant gender differences were detected in any multivariable model.

**Conclusion:**

In this surgically treated cohort from a dry-hot valley region, cataracts presented with a disproportionately high prevalence of severe opacities, particularly posterior subcapsular cataract, with evidence of early onset. The findings are hypothesis-generating and suggest that environmental factors, including intense UV exposure, may contribute. However, individual-level exposure data are required to confirm causality. These results support the adoption of age-stratified cataract screening strategies and UV protection measures in ecologically vulnerable regions with similar environmental characteristics.

## 1 Introduction

Studies indicate that as of 2020, cataracts remain the leading cause of blindness and visual impairment globally among individuals aged 50 and older. Cataract-related blindness affects over 15 million people, accounting for 45% of the global total of 33.6 million blind individuals. It has a notably high prevalence in developing countries (1). Ultraviolet (UV) exposure and the prevalence of age-related cataracts increase at lower latitudes and higher altitudes (2). The incidence of cataracts in areas with an average daily sunlight exposure of 12 h is 3.8 times higher than that in regions with only 7 h of exposure, and this figure continues to rise due to ozone layer depletion and increased UV radiation (3). Derong County, located in the Ganzi Tibetan Autonomous Prefecture at the westernmost edge of Sichuan Province (coordinates 28°10′ to 29°20′ N, 99°07′ to 99°34′ E), has an average altitude of 2,900 meters, exhibits a rare dry-hot valley climate, including annual average sunshine > 2,500 hours, total solar radiation ≥ 6,000 MJ/m², low humidity, and strong wind and sand. While high-altitude cataracts have been studied in Tibet and the Himalayas (4), no investigation has specifically characterized lens opacities in a dry-hot valley ecosystem, where the combination of UV reflection off exposed rock and sand, high temperature, and arid conditions may uniquely influence cataract phenotype. This study provides a descriptive analysis of cataract types and severity in a consecutive series of surgical series from this unstudied population, examines the associations between cataract characteristics and the demographic factors age and gender, and puts forward research hypotheses for future regional environmental and public health studies. To the best of our knowledge, this is the first report on cataract characteristics from a high-altitude dry-hot valley. It provides baseline data that can inform the development of region-specific prevention strategies and serves as a reference for similar ecologically vulnerable regions worldwide.

## 2 Methods

This retrospective descriptive case series included all patients who underwent cataract surgery or were listed for surgery at the People's Hospital of Derong from October 1, 2023, to June 20, 2025. The inclusion criterion was a diagnosis of age-related cataracts in at least one eye that met the surgical criteria confirmed by slit-lamp examination. Patients were aged 35 to 90 years at the time of surgery. The exclusion criteria were as follows: traumatic cataracts, complicated cataracts, congenital cataracts, anterior segment diseases, and severe systemic diseases.

Demographic data, including age, gender, occupation, township of residence, and medical history, as well as ophthalmic examinations, including visual acuity, slit-lamp examination, fundus photography, optical coherence tomography (OCT), and corneal endothelium examination, were extracted from electronic medical records. All patients were Tibetan farmers or herders with long-term local residence. Surgical indication was defined as best-corrected visual acuity (BCVA) ≤ 20/40 (Snellen equivalent) in the affected eye, with lens opacity confirmed via slit-lamp examination, or patient-reported visual dysfunction severe enough to interfere with daily activities, which aligns with the Chinese clinical guidelines for cataract surgery.

All cataract surgeries were performed using the Geuder Megatron® phacoemulsification system (Geuder AG, Heidelberg, Germany). For surgical technique, either the stop-and-chop or divide-and-conquer approach was selected at the surgeon's discretion based on nuclear density. All procedures were conducted through clear corneal incisions, with continuous curvilinear capsulorhexis completed for all cases. Standard postoperative care consisted of topical antibiotics (levofloxacin 0.5%) and corticosteroids (prednisolone acetate 1%), which were tapered over four weeks. No severe intraoperative or postoperative complications requiring deviation from the standard protocol were recorded in this cohort.

Age-related cataracts are classified into three main types based on the location of the opacity: cortical cataract, nuclear cataract, and posterior subcapsular cataract (PSC). All study participants underwent pupillary dilation, and lens opacities were graded using the Lens Opacities Classification System III (LOCS III) (5) by two experienced cataract specialists. Any disagreements were resolved by a third senior specialist. Inter-rater reliability was high (Cohen's kappa = 0.86). All graders were masked to all demographic and clinical information except age, which is easily identifiable during the grading process.

Nuclear opacity (N) was graded from N1 to N6, cortical opacity (C) from C1 to C5, and posterior subcapsular opacity (P) from P1 to P5. In this cohort, neither N6 nor P5 was observed. The maximum nuclear opacity grade was N5, and the PSC was P4. Mature cataract was defined per individual eye as Grade 5 cortical opacity (C5) or Grade 4 posterior subcapsular opacity (P4), which presents with complete loss of the fundus red reflex during direct ophthalmoscopy.

The patients were categorized into five age groups: 35–50, 51–60, 61–70, 71–80, and 81–90 years old. All younger patients (≤50 years) underwent comprehensive medical history collection and slit-lamp examination to rule out secondary causes of cataract, including trauma, uveitis, corticosteroid use, and congenital anomalies, only patients without any identifiable secondary cause were enrolled in the study. The inclusion of patients as young as 35 years reflects the observation that age-related lens opacities meeting surgical criteria can develop at an earlier age in this region, a finding that merits independent investigation. Per the LOCS III standard photographic reference standards, we defined severe opacity as grades C4-5, N4-5, and P3-4, as these grades typically correspond to opacities that impair visual function to a degree that meets surgical indications in routine clinical practice.

Since cortical, nuclear, and posterior subcapsular opacities frequently coexist in the same eye, we analyzed each opacity type separately rather than assigning a single mutually exclusive subtype diagnosis to each eye. The data were analyzed to investigate the associations of age and gender with different grades of cortical, nuclear, and posterior subcapsular opacities. A chi-square test was used for categorical variables. Kendall's tau-b was used for age-grade correlation. The Mann–Whitney U test was used to analyze gender differences.

All primary analyses were conducted at the eye level, including 556 eyes from 330 patients. Among all enrolled patients, 226 (68.5%) provided both eyes, and 104 (31.5%) contributed only one eye. To adjust for inter-eye correlation, we applied patient-level clustering and used cluster-robust standard errors (the Huber-White sandwich estimator) in all multivariable logistic regression models. We employed complete-case analysis as all variables included in the models had no missing values. The Hosmer-Lemeshow goodness-of-fit test was applied to evaluate model fit.

In multivariable logistic regression, the dependent variables were severe cortical opacity (C4-5 vs. C1-3), severe nuclear opacity (N4-5 vs. N1-3), and severe PSC (P3-4 vs. P1-2). The independent variables were age (continuous), gender, diabetes, and hypertension. The number of outcome events for each model was as follows: 196 events for severe cortical opacity, 93 events for severe nuclear opacity, and 336 events for severe posterior subcapsular opacity. No missing data were detected for any of the variables included in the regression models (age, gender, hypertension, and diabetes), thus no imputation methods were needed.

To address potential bias from including both eyes of the same patient, we performed a secondary analysis using one randomly selected eye per patient (330 eyes). Results are provided in Supplementary Table S1. All analyses were performed using SPSS v27.0. A *p* < 0.05 was considered statistically significant. Given the descriptive and hypothesis-generating nature of the study, *p*-values are interpreted as descriptive measures of evidence, not confirmatory ones.

## 3 Results

### 3.1 Baseline characteristics

The study included 556 eyes from 330 patients, with a mean age of 70.18 ± 10.47 years, ranging from 35 to 90 years. Among these eyes, 292 eyes (52.52%) were from females. The age distribution was as follows: 35–50 years: 20 eyes (3.60%); 51–60 years: 76 eyes (13.67%); 61–70 years: 126 eyes (22.66%); 71–80 years: 253 eyes (45.50%); 81–90 years: 81 eyes (14.57%). The mean presenting visual acuity (logMAR) was 1.12 ± 0.45, ranging from 0.30 to 2.30, and the mean BCVA (logMAR) was 0.85 ± 0.38, ranging from 0.18 to 1.70. Among the 556 included eyes, 482 (86.7%) had BCVA ≤ 20/40, which met the surgical indication threshold. Among the patients, 15 (4.5%) had diabetes, 110 (33.3%) had hypertension, and 9 (2.7%) had both conditions. It should be noted that these categories are mutually exclusive, and patients in the comorbidity group are not counted in the single-disease groups. Baseline characteristics are summarized in Table 1.

**Table 1:** Baseline characteristics of the study population (*N* = 330 patients, 556 eyes).

Variable	Value
Age (years), Mean ± SD (Min-Max)	70.18 ± 10.47 (35–90)
Age group (years)-*n* (%) of eyes	
35–50	20 (3.60%)
51–60	76 (13.67%)
61–70	126 (22.66%)
71–80	253 (45.50%)
81–90	81 (14.57%)
Gender (by eyes)-*n* (%)	
Male	264 (47.48%)
Female	292 (52.52%)
Comorbidities (by patients)-*n* (%)	
Hypertension	110 (33.3%)
Diabetes	15 (4.5%)
Both hypertension and diabetes	9 (2.7%)
Total eyes	556

Hypertension, diabetes, and comorbidity of the two are mutually exclusive categories. Patients with both conditions are not counted in the single-disease hypertension or diabetes categories.

### 3.2 Distribution of cataract opacity types and severity

The distributions of cortical, nuclear, and posterior subcapsular opacity grades by age group and gender are shown in Tables 2–4. Aligning with our analytical framework, we examined each opacity type independently. Cortical opacity had the highest mean grade (3.18), followed by posterior subcapsular opacity (2.64) and nuclear opacity (2.59). Severe opacities (C4-5, N4-5, P3-4) accounted for 35.3%, 16.7%, and 60.4% of eyes, respectively. The most frequent individual grade was C2 (32.9%) for cortical opacity, N2 (49.6%) for nuclear opacity, and P3 (38.5%) for posterior subcapsular opacity.

**Table 2:** Distribution of cortical opacity grade by age group and gender (*N* = 556 eyes).

Variable	Grade 1 (*n*, %)	Grade 2 (*n*, %)	Grade 3 (*n*, %)	Grade 4 (*n*, %)	Grade 5 (*n*, %)	Chi-square (*P*)
Age group (years)						39.45 (<0.001)
35–50 (*n* = 20)	3 (15)	7 (35)	0 (0.0)	3 (15)	7 (35)	
51–60 (*n* = 76)	2 (2.6)	31 (40.8)	15 (19.7)	10 (13.2)	18 (23.7)	
61–70 (*n* = 126)	9 (7.1)	45 (35.7)	42 (33.3)	11 (8.7)	19 (15.1)	
71–80 (*n* = 253)	2 (0.8)	80 (31.6)	76 (30)	33 (13)	62 (24.5)	
81–90 (*n* = 81)	3 (3.7)	20 (24.7)	25 (30.9)	14 (17.3)	19 (23.5)	
Gender						9.57 (0.0483)
Male (*n* = 264)	15 (5.7)	88 (33.3)	77 (29.2)	32 (12.1)	52 (19.7)	
Female (*n* = 292)	4 (1.4)	95 (32.5)	81 (27.7)	39 (13.4)	73 (25)	
Total (*N* = 556)	19 (3.4)	183 (32.9)	158 (28.4)	71 (12.8)	125 (22.5)	

**Table 3:** Distribution of nuclear opacity grade by age group and gender (*N* = 556 eyes).

Variable	Grade 1 (*n*, %)	Grade 2 (*n*, %)	Grade 3 (*n*, %)	Grade 4 (*n*, %)	Grade 5 (*n*, %)	Chi-square (*p*)
Age group (years)						119.74 (<0.001)
35–50 (*n* = 20)	4 (20.0)	16 (80.0)	0 (0.0)	0 (0.0)	0 (0.0)	
51–60 (*n* = 76)	10 (13.2)	57 (75.0)	6 (7.9)	3 (3.9)	0 (0.0)	
61–70 (*n* = 126)	18 (14.3)	67 (53.2)	30 (23.8)	6 (4.8)	5 (4.0)	
71–80 (*n* = 253)	5 (2.0)	109 (43.1)	91 (36.0)	36 (14.2)	12 (4.7)	
81–90 (*n* = 81)	0 (0.0)	27 (33.3)	23 (28.4)	21 (25.9)	10 (12.3)	
Gender						5.07 (0.2805)
Male (*n* = 264)	22 (8.3)	130 (49.2)	75 (28.4)	25 (9.5)	12 (4.5)	
Female (*n* = 292)	15 (5.1)	146 (50)	75 (25.7)	41 (14.0)	15 (5.1)	
Total (*N* = 556)	37 (6.7)	276 (49.6)	150 (27.0)	66 (11.9)	27 (4.9)	

**Table 4:** Distribution of posterior subcapsular opacity grade by age group and gender (*N* = 556 eyes).

Variable	Grade 1 (*n*, %)	Grade 2 (*n*, %)	Grade 3 (*n*, %)	Grade 4 (*n*, %)	Chi-square (*P*)
Age group (years)					42.98 (<0.001)
35–50 (*n* = 20)	3 (15.0)	2 (10.0)	8 (40.0)	7 (35.0)	
51–60 (*n* = 76)	15 (19.7)	15 (19.7)	25 (32.9)	21 (27.6)	
61–70 (*n* = 126)	35 (27.8)	27 (21.4)	47 (37.3)	17 (13.5)	
71–80 (*n* = 253)	36 (14.2)	65 (25.7)	95 (37.5)	57 (22.5)	
81–90 (*n* = 81)	13 (16.0)	9 (11.1)	39 (48.1)	20 (24.7)	
Gender					4.44 (0.3496)
Male (*n* = 264)	53 (20.1)	58 (22.0)	105 (39.8)	48 (18.2)	
Female (*n* = 292)	49 (16.8)	60 (20.5)	109 (37.3)	74 (25.3)	
Total (*N* = 556)	102 (18.3)	118 (21.2)	214 (38.5)	122 (21.9)	

Across age groups, cortical opacity consistently showed the highest mean grade (ranging from 2.9 to 3.5), with the highest mean value detected in the 81–90-year-old group (3.32), followed closely by the 71–80-year-old group (3.29). Nuclear opacity increased progressively with age, reaching a mean grade of approximately 3.2 in the 81−90-year-old group. Posterior subcapsular opacity exhibited elevated mean grades in the youngest (35−50 years) and oldest (81–90 years) age groups.

Age was significantly associated with higher grades for all three opacity types (cortical opacities: Kendall's tau-b = 0.336, *P* < 0.001; nuclear opacities: Kendall's tau-b = 0.432, *P* < 0.001; posterior subcapsular opacities: Kendall's tau-b = 0.187, *P* = 0.004). Gender was associated with cortical opacity grade (*P* < 0.05) but not with nuclear or posterior subcapsular grades (*P* > 0.05). In raw descriptive comparisons, females had slightly higher mean grades for cortical (3.3 vs. 3.1 in males) and posterior subcapsular opacities (2.7 vs. 2.5 in males). However, these differences were not significant in the multivariable analysis.

Severe posterior subcapsular opacity (P3-4) was the most common severe type overall (60.4%). In the young and middle-aged population (≤60 years, 96 eyes), severe posterior subcapsular opacity accounted for 61 eyes (63.5%). Mature cataracts were present in 141 eyes (25.4% of all eyes). Of these 141 eyes with mature cataracts, 26 (18.4%) were from patients aged ≤60 years; within the ≤60-year age subgroup, mature cataracts accounted for 27.1% (26/96 eyes). Age-related trends in mean grades are illustrated in Figure 1.

**Figure 1:**
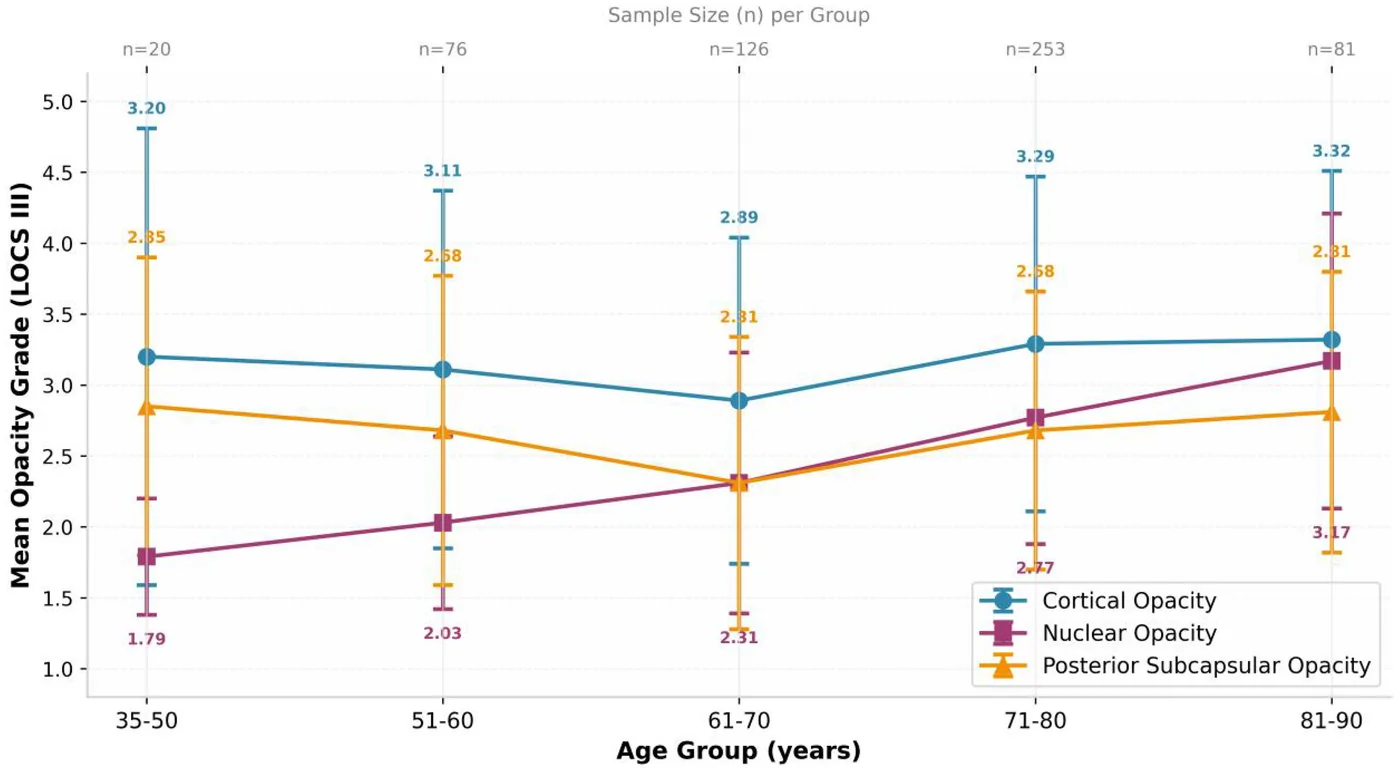
Mean opacity grade (±SD) of cortical, nuclear, and posterior subcapsular opacities across five age groups in patients undergoing cataract surgery (*N* = 556 eyes).

### 3.3 Multivariable logistic regression analysis

Multivariable logistic regression was performed to identify factors independently associated with severe cortical opacity (C4-5 vs. C1-3), severe nuclear opacity (N4-5 vs. N1-3), and severe posterior subcapsular opacity (P3-4 vs. P1-2). The model included age (continuous), gender, hypertension, and diabetes as covariates, with cluster robust standard errors to account for inter-eye correlation. The Hosmer-Lemeshow test indicated adequate model fit for all three models (cortical: *χ*^2^ = 5.64, *P* = 0.687; nuclear: *χ*^2^ = 4.92, *P* = 0.766; PSC: *χ*^2^ = 7.18, *P* = 0.517).

Age was a significant independent predictor for all three cataract types, with the strongest effect observed for nuclear opacity (OR per year = 1.101, 95%CI: 1.067–1.136, *P* < 0.001). Diabetes was significantly associated only with severe cortical opacity (OR = 2.313, 95%CI: 1.149–4.656, *P* = 0.019). Gender and hypertension were not independently associated with any severe cataract subtype (all *P* > 0.05). Detailed results are presented in Table 5.

**Table 5:** Multivariable logistic regression analysis of factors associated with severe cataract subtypes.

Variable	Severe cortical opacity (C4–5)		Severe nuclear opacity (N4–5)		Severe posterior subcapsular opacity (P3–4)	
	OR (95% CI)	*P*	OR (95% CI)	*P*	OR (95% CI)	*P*
Age (per year)	1.079 (1.051–1.107)	<0.001	1.101 (1.067–1.136)	<0.001	1.035 (1.011–1.059)	0.004
Gender (male vs. female)	0.917 (0.687–1.223)	0.554	0.877 (0.581–1.324)	0.534	0.758 (0.559–1.029)	0.075
Hypertension (yes vs. no)	1.156 (0.850–1.572)	0.351	1.328 (0.844–2.091)	0.222	0.997 (0.726–1.369)	0.986
Diabetes (yes vs. no)	2.313 (1.149–4.656)	0.019	0.878 (0.407–1.893)	0.739	1.309 (0.732–2.339)	0.363

Models were adjusted for all variables in the table. Cluster-robust standard errors (Huber-White sandwich estimator) were used to account for inter-eye correlation (patients with both eyes). Severe cortical opacity: grades 4–5 vs. 1–3; severe nuclear opacity: grades 4–5 vs. 1–3 (no grade 6 was observed); severe posterior subcapsular opacity: grades 3–4 vs. 1–2 (no grade 5 was observed). OR, odds ratio; CI, confidence interval.

Sensitivity analysis using one randomly selected eye per patient (*n* = 330 eyes) to account for inter-eye correlation yielded materially unchanged results (Supplementary Table S1).

## 4 Discussion

In this retrospective case series of 556 eyes from 330 patients who underwent cataract surgery in a dry-hot valley high-altitude region of western China, we found that although cortical opacity had the highest mean grade overall, posterior subcapsular opacity accounted for the highest proportion of severe opacities (60.4%). Notably, among patients aged ≤60 years, severe PSC was present in 63.5% of eyes, and mature cataracts were observed in 27.1% of this younger subgroup. Multivariable analysis showed that age was an independent risk factor for all three cataract subtypes, with the strongest effect observed for nuclear opacity. Diabetes was independently associated only with severe cortical cataract, while gender and hypertension were not significant predictors. A sensitivity analysis using one randomly selected eye per patient yielded materially unchanged results, confirming the robustness of our findings against inter-eye correlation.

Our finding that cortical opacity had the highest mean grade is consistent with reports from other high-altitude regions, such as the Tibetan Plateau (4). However, the extremely high proportion of severe PSC in patients younger than 60 years (63.5%) appears to exceed that reported in most previous studies. It is important to highlight that these findings are derived from a surgical cohort and therefore do not reflect the prevalence of cataracts in the general population. Instead, they characterize the disease burden among patients who have met the surgical indication for cataract extraction.

PSC has been linked to cumulative oxidative stress in previous experimental and epidemiological studies, particularly from UV radiation, which can damage lens epithelial cells at the posterior pole (6, 7). The dry-hot valley environment, characterized by intense UV reflection from exposed rocks and sand, over 2,500 annual sunshine hours, low humidity, and frequent sandstorms, could theoretically accelerate this process. Nevertheless, since we did not measure individual UV exposure or relevant lifestyle factors, we cannot confirm a causal association. Our observations are best described as hypothesis-generating. We propose these environmental hypotheses with caution, recognizing that the absence of individual-level exposure data means that further studies using objective dosimetry and well-controlled population cohorts are required to verify any causal relationship.

In our study, age was significantly associated with all three cataract types, which aligns with the well-established role of cumulative protein damage and oxidative stress (8, 9). The age effect was strongest for nuclear cataract, consistent with the slow, progressive aggregation of lens proteins. The weaker, though still significant, age effect on PSC suggests that non-age factors (such as UV or occupational exposure) may contribute more to PSC than to nuclear cataract in this population. Although unadjusted descriptive data showed slightly higher mean grades in females for cortical and PSC, gender was not an independent predictor in the multivariable model. This indicates that any apparent gender difference observed in the descriptive analysis is largely explained by age and comorbidities, rather than by gender itself.

We found an independent association between diabetes and severe cortical cataract, which is consistent with the known pathophysiology: hyperglycemia leads to sorbitol accumulation in the lens, inducing osmotic stress and subsequent damage to cortical fibers (10). The absence of a similar association with nuclear cataract or PSC further supports this subtype-specific relationship. However, since only 15 patients (4.5%) in this cohort had diabetes, this finding should be interpreted with caution and requires validation in larger, more diverse populations.

All patients were long-term residents of Derong County, a typical dry-hot valley characterized by high altitude, intense UV radiation, elevated temperatures, and arid conditions. The “Sun Valley” effect, prolonged sunshine, thin and dry air that reduces UV absorption, and high UV reflectance from the terrain result in a much higher cumulative UV dose than in lowland areas. Studies have indicated a positive correlation between the prevalence of cataracts and UV exposure (11–14). In addition, sustained high temperatures can alter the conformation of *α*-crystallins, interfering with protein stability (15, 16). Previous studies have shown that UV-B is strongly associated with cortical cataract (17–22), while UV-A contributes to PSC through oxidative damage to lens epithelial cells (23). Furthermore, high-altitude hypoxia may impair lens epithelial cell metabolism and antioxidant defenses (24, 25). The local diet, which is typically high in fat and low in vegetables and fruits, may increase lens lipid content and promote opacification (26).

We emphasize that our study cannot quantify the contribution of UV or other environmental factors owing to the lack of individual exposure data. Consequently, any discussion of environmental influences remains speculative, and these hypotheses require verification in future studies that incorporate objective exposure assessment, individual-level data collection, and comparative analyses with low-altitude populations. The environmental hypotheses proposed herein are intended to guide future research, rather than to establish definitive causal relationships.

Since only surgical cases were included, the proportion of severe opacities, particularly mature cataracts, is artificially inflated relative to the general population. The actual community distribution probably encompasses milder and earlier-stage cataracts that were not captured in this series. Despite the selection bias, our findings have practical implications for this underserved region. The high proportion of mature cataracts and the presence of advanced PSC in patients under 60 suggest that many individuals present late for surgical care. Barriers likely include distance to eye-care services, low awareness, and economic constraints.

Based on our descriptive findings, we propose several region-specific strategies for consideration by local health authorities. These are presented as potential approaches requiring prospective evaluation, rather than evidence-based recommendations that have been directly tested in this study. For example, we suggest annual eye examinations could be initiated at age 40 for local farmers and herders, with intensified monitoring for individuals aged 61 to 70 residing in dry-hot valleys, since this age group accounted for the highest proportion of surgical interventions in our study cohort. UV-400-certified sunglasses and wide-brimmed hats could be distributed through local health centers and agricultural cooperatives as a primary prevention measure, although the effectiveness of this intervention in this specific population requires prospective evaluation. Village health workers could be trained to educate communities on recognizing early PSC symptoms (e.g., glare, difficulty seeing in bright light) and on the importance of timely surgery before cataracts reach maturity. Periodic outreach surgical camps could also help reduce the backlog of mature cataracts and facilitate earlier surgical intervention. While these recommendations are not novel, they are tailored to a previously unstudied ecological setting and may serve as a reference for similar regions worldwide.

Our finding that severe PSC predominates among younger patients differs from the results of several previous studies. In the Beijing Eye Study, nuclear cataract was the predominant type in the general population (27). Similar to our observations, cortical cataract was the most prevalent type in the Tibetan Plateau study, however, the proportion of severe PSC was lower in that investigation (4). The high prevalence of PSC among younger patients in our cohort may reflect the unique environmental conditions of dry-hot valleys, although this hypothesis remains speculative in the absence of individual exposure data. Moreover, since our study did not include a low-altitude control group, we cannot determine whether these patterns are truly attributable to altitude or to other factors specific to dry-hot valleys.

## 5 Conclusion

This study reports on the characteristics of cataracts in a surgical cohort from a unique high-altitude dry-hot valley population. We observed that cataracts frequently present with severe posterior subcapsular opacities, particularly in patients aged ≤60 years, and that mature cataracts are common. Age was the strongest independent risk factor for all three cataract subtypes, and diabetes was specifically associated with severe cortical cataract. The high proportion of severe PSC in younger patients is consistent with the hypothesis that intense ultraviolet exposure may accelerate cataract development. However, this hypothesis remains unproven owing to the lack of individual UV exposure data and the absence of a control group. For public health initiatives in this region and similar settings, we suggest considering age-stratified cataract screening starting from age 40, intensified monitoring for moderate to severe lens opacities in the 61–70 age group, and prioritization of surgical indication assessments for individuals older than 71. We also suggest UV protective measures for outdoor workers and health education to promote earlier surgery. These suggestions require prospective evaluation before they can be recommended as evidence-based interventions. Future population-based studies incorporating objective UV dosimetry, longitudinal follow-up, comparison with low-altitude regions, and comprehensive adjustment for confounding factors are needed to establish causality and to guide evidence-based public health policy.

## 6 Limitations

This study has several important limitations that should be considered when interpreting the results. Firstly, selection bias is inherent to our design; as we only included surgical patients and individuals who met surgical criteria, our cohort naturally overrepresents more advanced cataract cases. Accordingly, the observed distribution of cataract types and severity cannot be generalized to represent cataract prevalence or phenotypes in the broader regional population, and we have explicitly noted this limitation throughout the manuscript. Secondly, the lack of a control group (e.g., low-altitude or non-dry-hot valley regions) prevents us from drawing causal inferences linking cataracts to environmental factors. Moreover, we did not collect individual-level data on UV exposure, occupational exposure, lifestyle factors, or dietary information, so our environmental speculation is purely for hypothesis generation.

In this study, we accounted for inter-eye correlation using cluster-robust standard errors in the primary analysis, and we additionally conducted a secondary single-eye analysis that yielded materially unchanged results. Even so, the primary per-eye results still require descriptive interpretation. Due to data limitations, potential confounders, including smoking status, medication exposure (particularly corticosteroid use), occupational history, socioeconomic status, nutritional status, and length of local residence, were not incorporated into the regression models. LOCS III grading system is semi-quantitative and is susceptible to both intra-observer and inter-observer variability, even though inter-rater reliability was high in our study. The single-center cross-sectional design limits the generalizability of our findings and precludes assessment of disease progression. With only 15 patients (4.5%) diagnosed with diabetes, the stability of the diabetes-related effect estimate in the regression model needs to be carefully considered and confirmed in larger-scale studies. Despite these limitations, this study provides valuable baseline data for an understudied population and highlights the urgent need for early detection and prevention in ecologically fragile high-altitude valleys.

## Data Availability

The original contributions presented in the study are included in the article/Supplementary Material, further inquiries can be directed to the corresponding author/s.
