# Supplementary material for: Characteristics of cataracts in dry-hot valley-type high-altitude surgical cohort: a retrospective descriptive case series of 556 eyes from western China
Source: Front Med (Lausanne). 2026 Sep 23;13:1921180. doi: 10.3389/fmed.2026.1921180 (PMC13642011; doi:10.3389/fmed.2026.1921180)
Supplement: Supplementary file 1 [file Table1.docx]

**Supplementary Materials**

**Table S1. Sensitivity Analysis: Multivariable Logistic Regression Using One Randomly Selected Eye per Patient (N = 330 eyes)**

| **Variable** | **Severe Cortical Opacity (C4–5)** | | **Severe Nuclear Opacity (N4–5)** | | **Severe Posterior Subcapsular Opacity (P3–4)** | |
| --- | --- | --- | --- | --- | --- | --- |
|  | **OR (95% CI)** | **P** | **OR (95% CI)** | **P** | **OR (95% CI)** | **P** |
| Age (per year) | 1.082 (1.048–1.117) | <0.001 | 1.104 (1.062–1.148) | <0.001 | 1.038 (1.006–1.071) | 0.018 |
| Gender (male vs. female) | 0.904 (0.626–1.306) | 0.591 | 0.861 (0.518–1.432) | 0.565 | 0.732 (0.491–1.092) | 0.124 |
| Hypertension (yes vs. no) | 1.168 (0.790–1.727) | 0.433 | 1.341 (0.763–2.359) | 0.306 | 1.015 (0.678–1.519) | 0.943 |
| Diabetes (yes vs. no) | 2.401 (1.025–5.627) | 0.044 | 0.903 (0.336–2.427) | 0.840 | 1.386 (0.632–3.042) | 0.418 |

Notes: Cluster-robust standard errors were not required for this analysis as each patient contributed only one eye.
